# RNA 3D Structure Prediction: Progress and Perspective

**DOI:** 10.3390/molecules28145532

**Published:** 2023-07-20

**Authors:** Xunxun Wang, Shixiong Yu, En Lou, Ya-Lan Tan, Zhi-Jie Tan

**Affiliations:** 1 Department of Physics, Key Laboratory of Artificial Micro & Nano-Structures of Ministry of Education, School of Physics and Technology, Wuhan University, Wuhan 430072, China; 2 School of Bioengineering and Health, Wuhan Textile University, Wuhan 430200, China; 3 Research Center of Nonlinear Science, School of Mathematical and Physical Sciences, Wuhan Textile University, Wuhan 430200, China

**Keywords:** RNA 3D structure, ensemble prediction, structure evaluation, structure refinement

## Abstract

Ribonucleic acid (RNA) molecules play vital roles in numerous important biological functions such as catalysis and gene regulation. The functions of RNAs are strongly coupled to their structures or proper structure changes, and RNA structure prediction has been paid much attention in the last two decades. Some computational models have been developed to predict RNA three-dimensional (3D) structures in silico, and these models are generally composed of predicting RNA 3D structure ensemble, evaluating near-native RNAs from the structure ensemble, and refining the identified RNAs. In this review, we will make a comprehensive overview of the recent advances in RNA 3D structure modeling, including structure ensemble prediction, evaluation, and refinement. Finally, we will emphasize some insights and perspectives in modeling RNA 3D structures.

## 1. Introduction

RNAs are a kind of fundamental biological macromolecule, in addition to proteins and DNAs. Numerous studies have demonstrated that RNA has many essential biological functions, such as regulating gene expressions [1], protein biosynthesis regulations [2], and catalytic biological reactions [3]. Furthermore, the multi-functional nature of RNAs significantly contributes to the design of related nanoscale biomedical and technological applications [4]. Generally, RNAs need to fold into their specific 3D structures to perform their specific functions [5,6,7]. For instance, ribozymes can only catalyze reaction functions when they fold into their native 3D structures [6], while riboswitches regulate gene expressions through dynamic changes in the 3D structures upon metabolite binding [5,7]. Therefore, the comprehensive knowledge of RNA 3D structures is of great significance for understanding and utilizing RNA biological functions.

Until now, experimental methods such as X-ray crystallography, nuclear magnetic resonance spectroscopy, and cryo-electron microscopy have been predominantly used to derive RNA 3D structures [8]. However, these methods are generally time-consuming and laborious, especially for RNAs with long sequences or complex structures [9]. Thus, the RNA 3D structures deposited in Protein Data Bank (PDB) [8] are still very limited relative to the vast number of RNA sequences stored in the central RNA [10] and the large number of 3D structures of proteins [8,9,10,11]. For example, there are only 1732 isolated RNA structures in the PDB database, while there are 34 million RNA sequences in the RNA central and 188,726 3D structures of proteins in the PDB database (until 23 May 2023). Hence, it is essentially necessary to develop computational models for modeling RNA 3D structures at a high resolution. 

In recent decades, various computational models have been developed to predict RNA 3D structures [11,12,13,14,15], and the models are generally composed of three procedures: predicting 3D structure ensembles, identifying near-native structures that are close to the experimentally determined native structures through a reliable scoring function/statistical potential, and refining identified near-native structures [11,15]. Here, we will go through them.

**Figure 1:**
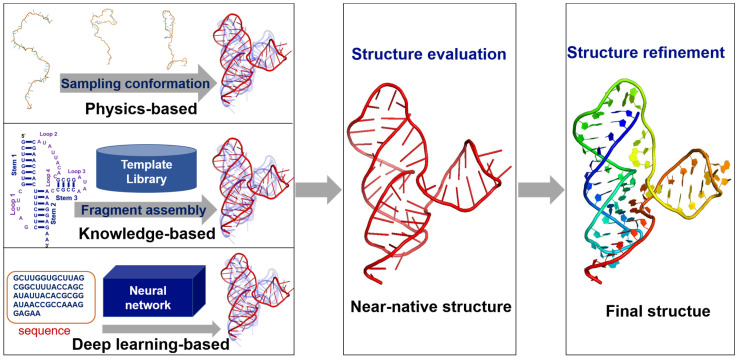
The general workflow for modeling RNA 3D structures: structure generation, structure evaluation, and structure refinement. There are three types of predictive models for RNA 3D structures, namely physics-based, knowledge-based, and deep-learning-based models.

First, a computational model generates an ensemble of RNA 3D structure candidates based on input information, such as RNA sequences solely or RNA sequences and secondary structures for some models [15]. For improving the prediction accuracy, some models can also involve additional constraint information, such as experimental information and the distance between specific atom pairs. Through physics-based force field and conformation sampling, or knowledge-based fragment assembly, or deep-learning-based information, a computational model can generate a structure candidate ensemble with near-native structures. Moreover, generally, these prediction models can generate a structure ensemble for different types of RNA structures based on sequence information or secondary structure information, such as hairpins, hairpins/duplexes with bulges/internal loops, pseudoknots, kissing complexes, and multi-way junctions. However, it is still difficult for existing models to predict the 3D structures of RNA G-quadruplexes from their sequences [11,15], while some models can be used to study RNA G-quadruplexes [16], i.e., HiRE-RNA can almost stabilize the native 3D structure of an RNA G-quadruplexes for 3 µs and a convolutional neural network-based model can identify potential RNA G-quadruplexes from transcriptomics data [17,18]. Moreover, some computational models display a competitive performance in RNA-Puzzles [19,20,21,22,23] and CASP-RNA [24]. Here, RNA-Puzzles is a CASP-like competition for RNA 3D structure prediction [19,20,21,22,23], and CASP-RNA is a prediction competition for RNA 3D structures newly present in CASP15 [24]. Depending on the methods for generating the 3D structure ensemble, the existing computational models can be roughly divided into physics-based [25,26,27,28,29,30,31,32,33,34,35,36,37,38,39,40,41,42,43], knowledge-based fragment assembly [44,45,46,47,48,49,50,51,52,53,54,55,56], and deep-learning-based ones [57,58,59,60]; see Figure 1. 

Second, a structure evaluation is required to identify the top near-native structures from the precedingly predicted 3D structure candidate ensemble [61,62], and a high-quality scoring function/energy function for structure evaluation should be able to identify the candidate structures closest to the native ones in the predicted ensemble [61,62]. For a blind prediction of an RNA 3D structure, a reliable scoring function/statistical potential is definitely required as the native structure of the target RNA is not available. These scoring functions/energy functions are generally developed based on the native structures in the PDB database, which would contain the major structure features of the native structures and would provide a reliable structure evaluation to identify the top structures in the predicted structure ensemble, even if the native structures are not available, and the existing scoring functions/energy functions for RNA 3D structure evaluation typically include physics-based energy functions [26], knowledge-based statistical potentials [63,64,65,66,67,68,69], deep-learning-based scoring functions [70,71], and clustering-involved algorithms [36,37].

Finally, structure refinement may need to be performed for the identified top structures so as to obtain high-quality RNA 3D structures as the structures may contain some unreasonable defects, such as unnatural bonds and serious conflicts between atoms [72,73,74]. In addition to modifying the local defects in RNA structures, a good refinement approach can also improve the global 3D RNA structures [73,75], making the structures to be overall closer to their native ones.

In this review, we provide a comprehensive overview of the recent advances in modeling RNA 3D structures, including structure ensemble prediction, structure evaluation, and structure refinement. The main text is organized as follows. First, we give a detailed overview of the existing RNA 3D structure prediction models, including physics-based, knowledge-based, and deep-learning-based models. Second, we describe the recent progress in RNA 3D structure evaluation. Third, we introduce recently developed methods for RNA 3D structure refinement. Finally, we discuss the challenges in modeling RNA 3D structures and offer some insights in modeling RNA 3D structures.

## 2. RNA 3D Structure Prediction Models

### 2.1. Physics-Based Models

**Figure 2:**
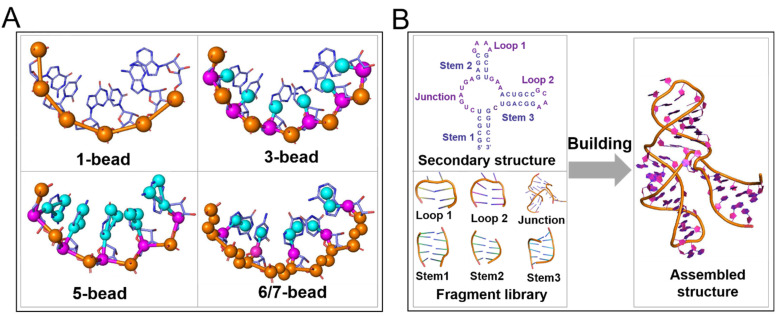
(**A**) Major coarse-grained (CG) representations for existing physics-based models. (**B**) A typical schematic diagram for building an RNA 3D structure through knowledge-based fragment assembly models.

The physics-based models are based on the fundamental physics principle that the native structures are those states with the lowest energies for RNAs [15]. In principle, all-atom molecular dynamics simulations such as Amber [76,77,78] and Charmm [79,80] can be used to predict RNA 3D structures, while due to the huge computation from the all-atom representation of RNAs, solvents, and ions, such all-atom-based simulation methods are only limited to very small RNAs with very simple topological structures such as duplex and hairpin [81]. To reduce the atom-representation-concerned complexity, some physics-based coarse-grained (CG) models with varying CG levels have been developed to predict RNA 3D structures in a reasonable time [25,26,27,28,29,30,31,32,33,34,35,36,37,38,39,40,41,42]. The typical CG representations are illustrated in Figure 2, and the existing physics-based CG models are summarized in Table 1, as well as the corresponding references and the available websites [25,26,27,28,29,30,31,32,33,34,35,36,37,38,39,40,41,42]. Generally, a physics-based CG model guides RNA structure folding through a specific force field (energy function) and a conformation sampling algorithm such as Monte Carlo (MC) [82] or molecular dynamics (MD) sampling [83,84]. A CG force field in these models is generally composed of bonded and non-bonded energy terms [30,31,32,33,34,35].

(1) $$E=E_{\mathrm{bond}}+E_{\mathrm{angle}}+E_{\mathrm{dihedral}\;\mathrm{angle}}+E_{\mathrm{exclusion}\;\mathrm{volume}}+E_{\mathrm{base}\;\mathrm{paring}}+E_{\mathrm{base}\;\mathrm{stacking}},$$

where the bonded energy term includes bond length, bond angle, and dihedral/torsion angle energies, and the non-bonded energy term usually includes exclusion volume, base pairing, and base stacking energies. In the following, we mainly introduce physics-based predictive models with different CG representations for RNA 3D structures rather than the all-atom molecular dynamic simulations.

#### 2.1.1. One-Bead Nucleotide Model

*YUP.* In the YUP model, a bead located at the P atom is used to represent a nucleotide. Through the one-bead energy function and MC sampling algorithm, YUP can be used to simulate RNA structure folding, and the structure with the lowest energy in the conformational ensemble is chosen as the predicted final structure [25,26]. Although the model may require additional experimental information such as a secondary structure to predict RNA 3D structures, YUP has been developed into an adaptive package for the automatic CG modeling of RNA structures. Furthermore, the model can also predict protein and DNA structures with additional constraint information. In addition, the YUP model can be found at http://rumour.biology.gatech.edu/YammpWeb/.

*NAST.* Similar to YUP [26], NAST is also a one-bead CG model, and a bead located at the C3′ atom is used to represent a whole nucleotide [27]. The model utilizes RNA-specific knowledge-based potential energy and MD algorithms to predict the RNA 3D structures, and the predicted structures can be obtained through the clustering method for 1500 low-energy conformations. By involving the secondary and tertiary contact information, NAST can predict the RNA 3D structures. Moreover, NAST predicts the 3D structures of the yeast phenylalanine tRNA (76 nt) and the P4-P6 domain of the Tetrahymena thermophile group I intron (158 nt) with ~8 Å and ~16 Å RMSDs from the native structures, respectively [26]. In addition, NAST can combine small-angle X-ray scattering data [85] and experimental solvent accessibility data as filters to rank the clusters of similar structures. The package of NAST can be downloaded at https://simtk.org/home/nast.

#### 2.1.2. Three-Bead Nucleotide Model

*iFoldRNA/iFoldRNA2.* In the iFoldRNA model, three beads are used to represent each nucleotide: the three beads are located at the mass center of a phosphate group, at the center of the five-atom ring of a sugar, and at the center of the six-atom ring of a base [28,29]. Moreover, the bonded terms are composed of bond length, bond angle, and dihedral angle interactions, and non-bonded terms are composed of base pairing, base stacking, short-range phosphate–phosphate repulsion, and hydrophobic interactions. Through the discrete MD algorithm and clustering method for low-energy conformations, iFoldRNA can predict RNA 3D structures from their sequences. iFoldRNA allows for the prediction of the structure of short RNAs (<50 nt) within 4 Å RMSD from the corresponding experimental structures. Additionally, iFoldRNA can predict complex RNAs by involving experimental information such as base-pairing and hydroxyl-radical probing. iFoldRNA has been developed into the iFoldRNA2 [29] webserver at http://ifoldrna.dokhlab.org. 

*A CG model with salt effect.* A three-bead CG model with a salt effect has been developed for predicting RNA 3D structures in the presence of monovalent/divalent ions [30,31,32,33,34,35] due to the polyanionic nature of RNAs [86,87,88]. In contrast, most existing 3D structure prediction models are generally focused on predicting the 3D structures of RNAs, and the effects of salt ions and temperature are seldom involved in the models. In the three-bead CG model with salt effect, the three beads are located at P, C4′, and N9 (N1) atoms of purine (pyrimidine), respectively. In the energy function of the model, the salt effect is implicitly involved through a combination of the counterion condensation theory [89] and the tightly bound ion model [90,91,92], and the effect of temperature is accounted for through involving the experimental parameters of base stacks from Turner et al. [93]. Using the MC simulated annealing or replica-exchange MC (REMC) algorithm [94], the model can predict 3D structures for RNA hairpins, kissing complexes, and minimal H-type pseudoknots and complex pseudoknots from sequences and the thermal stabilities of the RNAs in monovalent/divalent ion solutions, including SARS-CoV-2 programming-1 ribosomal frameshifting element and Zika virus xrRNA [30,31,32,33,34,35]. The mean RMSD of the predicted structures is less than 7 Å for RNAs (<~90 nt) [30,31,32,33,34,35]. In addition, the mean deviations of the melting temperatures from the experimental data are ~2 °C for RNA pseudoknots and kissing complexes [30,31,32,33,34,35] over extensive Na^+^/Mg^2+^ concentrations. However, the model still cannot make good predictions for RNAs with multi-way (≥3) junctions. Anyway, this model can capture the effects of salt and temperature on predicted RNA 3D structures, which are seldom captured by other existing RNA 3D prediction models.

#### 2.1.3. Five-Bead Nucleotide Model

*SimRNA.* In the SimRNA model, the five beads are located at the P atom, C4′ atom of the sugar, C2 atom, N1 atom, and C4 atoms of pyrimidine (N9 and C6 atoms of purine) [36,37]. The energy function of SimRNA comprises sequence-independent local-ranged terms and sequence-dependent long-ranged terms. The local terms consist of bond length, flat angle, and torsional angle interactions, and the long-ranged terms include base–base, base–backbone, and backbone–backbone interactions [36]. Using the REMC algorithm, SimRNA can predict RNA 3D structures solely from sequences in a reasonable time, and the predicted final structures are obtained by the clustering method for the low-energy conformations. Additionally, secondary structures or other constraint information can be added into SimRNA to improve the prediction accuracy and efficiency, especially for complex RNAs. SimRNA can effectively predict RNA with relatively short RNA sequences (< ~50 nt). However, for RNAs with longer sequences (>70 nt), the prediction accuracy of the model can be improved if the secondary structures are provided [36]. SimRNA has been developed into the versions of software package and webserver, which can be found at https://genesilico.pl/SimRNAweb.

*IsRNA.* In the IsRNA1 model, four/five beads are used to represent a nucleotide [38,39,40]. Specifically, two beads are located at the P atom of the phosphate group and the C4′ atom of sugar, and three/two beads are positioned at the center of mass of the constituent heavy-atom groups to represent purine/pyrimidine bases, respectively. The energy functions of IsRNA1 [39] were derived through an iterative simulated reference state approach [38,39], and the MD or REMD algorithm was employed to accelerate conformational sampling. In the model, the predicted final structures are obtained by a clustering method for the 10% conformations with the lowest energy in the candidate ensemble. IsRNA1 can predict the structures of small RNAs based on their sequences and more complex RNAs based on their secondary structures. IsRNA1 has been examined against a large-scale benchmark dataset (containing 40–161 nts) with secondary structure constraints. The dataset includes 44 stem loops, 43 multi-way junctions, and 43 structures of long-range tertiary interactions, and the mean RMSD of the predicted top-1 structure is less than 10 Å [39]. Moreover, the model can be used to optimize the structures generated by two fragment assembly methods [54,55]. Very recently, IsRNA was developed into IsRNA2 to predict 3D structures for RNAs with noncanonical base pairs [40], and the web server of IsRNA is available at http://rna.physics.missouri.edu/IsRNA/index.html.

*RNAJP.* Very recently, Li et al. developed a five-bead CG model called RNAJP to predict RNA 3D structures, especially multi-way junction structures [41]. Specifically, two CG beads are placed at the P and C4 atoms, representing the phosphate and sugar groups, and three beads are located at the N1/N9, C2, and C4/C6 atoms for pyrimidine/purine, respectively. In addition to the conventional bonded and non-bonded energy terms, similar to other physics-based CG models discussed above, the RNAJP model explicitly considers the interactions between adjacent helices and between adjacent strands in the junction structures and the long-ranged interactions between loops. With the use of the toolkit OpenMM [95], RNAJP can reliably predict RNA 3D structures with secondary structures as the input, and the top-1 structure predicted by RNAJP is identified through a specific energy function [41]. Although the model cannot predict RNA 3D structures solely from sequences, it can predict high-quality RNA 3D structures with given sequences and their corresponding secondary structures, especially for three- and four-way junctions, and the predictions of RNAJP can reach a mean RMSD of ~8 Å for the 22 RNA three-way junction structures (53–160 nts) and 5 RNA four-way junction structures (68–155 nts). However, the model cannot create reliable predictions for five-way or higher-way junctions due to the extremely high structural complexity [41]. The source code of the RNAJP model can be downloaded at http://rna.physics.missouri.edu/RNAJP/index.html.

#### 2.1.4. Six/Seven-Bead Nucleotide Model

*HiRE-RNA.* The HiRE-RNA model is a high-resolution CG model that uses six to seven beads to represent a nucleotide [42]. Specifically, five CG beads are assigned to the phosphate group and sugar rings (namely, P, O5′, C5′, C4′, and C1′ atoms), and another one/two beads are located at the mass centers of nonhydrogen atoms of the pyrimidine/purine base. In the force field of HiRE-RNA, harmonic form potentials were used to describe the bond length and bond angle energies in the bonded terms, while the torsional angle energy was described with a cosine form potential. Moreover, non-bonded terms include exclusion volume, electrostatic, base pairing, and base stacking potentials. Using the REMD algorithm [96] for sampling conformations and the clustering method for low-energy conformations, HiRE-RNA can predict RNA 3D structures from sequences and complex RNAs with secondary structure information, and HiRE-RNA has been tested on 13 RNAs (12–71 nts), and the RMSDs are less than ~8 Å from the corresponding native structures [42]. Additionally, HiRE-RNA can be used to examine the stability of RNA hairpins and duplexes, while the energy function of the model may require further optimization to improve the predictions for the thermodynamic properties of RNAs. 

#### 2.1.5. Coarse-Grain Helix-Centered Model

*Ernwin*. Unlike the above models, Ernwin is a helix-centered CG model, which uses one line segment and two vectors to represent a helix and considers elements (loops) linking helices as the degrees of freedom [43]. The energy function of Ernwin comprises five separate terms: two terms attributed to clash and junction closure, which serve as constraint energies to exclude impossible structures based on physical forces; the other three terms accounting for radius of gyration, A-minor energy, and loop–loop interaction energy serve as non-constraint energies, which are knowledge-based potentials derived from the known structures in the PDB database [8]. Combining the energy function with Markov chain Monte Carlo simulation, the Ernwin model can efficiently predict RNA 3D structures based on their secondary structures, and the structure with the lowest energy in the conformational ensemble is chosen as the predicted final structure. The package of the model is freely available at http://github.com/pkerpedjiev/ernwin.

As described above, an important advantage of various physics-based CG models is that they can predict RNA 3D structures solely from sequences based on different specific force fields and various MC/REMC or MD/REMD conformation sampling algorithms. However, even with such CG simplification for nucleotides, to predict the 3D structures of large RNA at high accuracy solely from sequences remains challenging for a physics-based CG model. The reliable predictions from the existing physics-based CG models are still limited to RNAs of medium size (<~90 nt with inputting only sequences or <~150 nt with inputting secondary structures). Namely, the accuracy of the CG force field and the conformation sampling algorithm are still required to be refined and developed to improve the prediction accuracy and efficiency. In addition, due to the polyanionic nature of RNAs, the structures and stabilities of RNAs are usually sensitive to solution ion conditions, especially to multivalent ions [90], while the effect of ions such as Mg^2+^ are rarely involved in the existing 3D structure prediction models. Although the effects of monovalent/divalent ions on RNA thermodynamics can be captured by the three interaction sites (TIS) model developed by Thirumalai et al. [97,98,99], the TIS model is a Gö-like model and cannot make predictions for RNA 3D structures from sequences. The CG model with salt effect proposed by Tan et al. can create reliable predictions for the thermal stabilities of (complex) RNA pseudoknots in monovalent/divalent ions [33,35], while it is still a challenge for this model to predict more complex RNA structures (e.g., multi-way junctions). It is necessary to develop a physics-based CG model to efficiently and reliably predict 3D structures and stabilities of complex RNA structures in monovalent/divalent ion solutions.

### 2.2. Knowledge-Based Fragment Assembly Models

As macromolecule 3D structures evolve much slower than their sequences, evolutionarily related macromolecules typically retain similar 3D structures, despite their differences at the sequence level [11,15]. Moreover, the number of experimental structures deposited in the PDB database increases gradually with the development of various experimental technologies [11]. Therefore, RNA 3D structures can be predicted through so-called knowledge-based methods [11,15]. The early knowledge-based methods were graphics-based methods such as MANIP [100] and RNA2D3D [101], which could intuitively and quickly predict large-scale RNA 3D structures, but required users to have professional knowledge of a high level. Later, some fully automated fragment-assembly methods were developed and widely employed for RNA 3D structure predictions [44,45,46,47,48,49,50,51,52,53,54,55,56]. Typically, the fragment assembly models predict RNA 3D structures based on RNA sequences and secondary structures as inputs. Figure 2 shows the typical schematic diagram for existing fragment assembly models, which can be roughly classified into two categories based on the size of the fragments used: small motifs as fragments and medium motifs as fragments. In the following, we introduce the existing knowledge-based, fully automated fragment assembly models for RNA 3D structures, which are summarized in Table 2.

#### 2.2.1. Small Motifs as Fragments

*FARNA/FARFAR/FARFAR2.* Das et al. proposed a fragment assembly model named FARNA/FARFAR for predicting RNA 3D structures based on three-nucleotide fragments [44,45]. FARNA/FARFAR assemble RNA 3D structures guided by the specific knowledge-based energy function and MC algorithm. As an early RNA 3D structure assembly model, FARNA/FARFAR has a good prediction accuracy for small-size RNAs. Later, Das et al. proposed FARFAR2 [46], in which four treatments were implemented to improve the prediction accuracy and efficiency, including (a) an updated fragment library, (b) a fractional filter for the fragment assembly process, (c) a special Monte Carlo movement, and (d) a new all-atom scoring function. Compared with FARFAR, FARFAR2 recovers near-native structures more accurately and predicts the 3D structures of adenovirus virus-associated RNA and five riboswitch complexes with RMSDs of ~3–14 Å [46]. FARFAR2 has been developed into a user-friendly webserver at https://rosie.rosettacommons.org/farfar2.

#### 2.2.2. Medium Motifs as Fragments

*MC-Fold/MC-Sym*. Parisien et al. proposed an RNA structure prediction pipeline consisting of two computational models: MC-Fold and MC-Sym [47]. In the pipeline, MC-Fold predicts RNA secondary structures, and then MC-Sym assembles an ensemble of 3D structures based on the secondary structures. Different thermodynamic models such as Mfold [103], MC-Fold can predict secondary structures for RNAs with noncanonical base pairs with the use of knowledge-based scoring functions based on NCM (nucleotide cyclic motif) databases. The NCM database contains lone-pair loops up to six nucleotides, including the flanking lone base pair, and double-stranded NCMs up to eight nucleotides including both flanking base pairs. MC-Sym performs the fragment insertion simulation with the 3D NCMs and the Las Vegas algorithm. The MC-Fold/MC-Sym pipeline has been tested by building 3D structures of precursor microRNA and proposing a new structure of the human immunodeficiency virus (HIV) cis-1 frameshift segment, and the model has been tested against 13 different types of RNAs (29–47 nts) with a mean RMSD of ~2 Å [47]. The web server of the MC-Fold/MC-sym pipeline can be found at http://www.major.iric.ca.

*RNAComposer.* Popenda et al. developed a fast and fully automated fragment assembly model named RNAComposer for predicting RNA 3D structures based on secondary structures [48,49]. The model employs smallest secondary elements (SSE) as blocks, which helps the model to achieve RNA 3D structure prediction at a relatively high accuracy. The predicted top-1 structure in RNAComposer is assembled from the best fragments, which are evaluated based on four criteria: secondary structure topology, sequence similarity, source structure resolution, and energy based on the Charmm force field. RNAComposer was examined for 40 different types of RNAs (31–161 nts), which include hairpins without/with internal loops, pseudoknots, and multi-way junctions; the mean RMSD of the top-1 structure is ~5 Å [48]. RNAComposer has been developed into a user-friendly webserver version at http://rnacomposer.ibch.poznan.pl.

*3dRNA.* Similar to RNAComposer [48], 3dRNA also employs SSEs as building blocks to predict RNA 3D structures [50,51,52,53], while in 3dRNA, the 3D structures of the SSEs extracted from the experimental structures contain one more base pair at their 5′-end for more accurate superposition between different fragments in the global structure assembly. Notably, the assembled 3D structures can be further optimized by a specific CG energy function with the information from direct coupling analysis [75]. In 3dRNA, the final predicted structure can be identified by the clustering algorithm for the assembled structures and a specific scoring function named 3dRNAscore [68] to rank the candidates in the different clusters. 3dRNA exhibits relatively high prediction accuracy for different types of RNA structures, particularly pseudoknots and large RNAs with tertiary contacts. 3dRNA has been tested against extensive RNAs including five very large RNAs (500–3000 nts), and it is encouraging that the predicted structures of four of the five large RNAs have good performance with RMSDs within 15 Å [52]. In addition, 3dRNA can predict the 3D structures of circular RNAs [53]. 3dRNA has been developed into a friendly webserver version at http://biophy.hust.edu.cn/new/3dRNA.

*Vfold3D/VfoldLA.* Cao et al. proposed two different fragment assembly models, namely Vfold3D [54] and VfoldLA [55]. With a given secondary structure, Vfold3D automatically assembles RNA 3D structures with the 3D fragments from the PDB database based on resolved secondary motifs such as hairpin loops and multi-way junction loops [54]. However, Vfold3D is limited by the small number of known RNA structures, especially for the templates of multi-way junctions. Xu et al. developed a new model of VfoldLA with template search and the assembly algorithm to build RNA 3D structures [55], where templates with single-stranded loops/junctions are searched for instead of whole motifs, and a whole multi-junction loop can be assembled based on single-stranded loops in the absence of such a whole multi-junction loop [55]. Furthermore, a hybrid method has been proposed that combines Vfold3D and VfoldLA to predict the RNA 3D structures [104]. The hybrid method is focused on the definition of motifs and loops, the processing of template-free motifs, and the 3D structure assembly based on motifs and loops templates [104]. Moreover, the Vfold-pipeline was developed by integrating Vfold2D (secondary structure prediction model), Vfold3D, and VfoldLA [105].The pipeline has been examined on 92 RNAs, including hairpin/internal loops, 3-, 4-, and 5-way junctions, and pseudoknots, and the mean RMSDs of the predicted structure are ~6 Å, ~10 Å, ~7 Å, ~15 Å, and ~16 Å, respectively [106]. Vfold3D/VfoldLA are available for users on their webservers, and the Vfold-pipeline can be found at http://rna.physics.missouri.edu/vfoldPipeline/index.html.

*FebRNA.* Very recently, Zhou et al. proposed a fragment-ensemble-based model named FebRNA for building RNA 3D structures with secondary structures as the input [56]. This model selects almost all of the templates according to the types of secondary motifs and the lengths, regardless of the sequences and transfers the all-atom fragments into CG ones according to the CG model with salt effect [35], resulting in a global CG 3D candidate ensemble with a vast number of assembled structures (up to ~25,000). This naturally increases the likelihood of including the structures very close to the native structures [56]. Moreover, when building the global structures, FebRNA prefers loop templates with more end base pairs, which helps to better describe the orientation of the stems and improves the accuracy of the final structures. The predicted structures are identified using a specific scoring function from an efficient CG scoring function of cgRNASP [67]. Afterwards, the identified top CG structures are rebuilt into all-atom structures. It has been demonstrated that FebRNA can reliably and efficiently predict the 3D structures of different types of RNAs, including 14 RNA hairpins (17–31 nts), 8 pseudoknots (28–127 nts), 25 multi-way junctions (54–393 nts), and 16 RNA-Puzzles (37–189 nts), and the mean RMSD of the predicted top-1 structures is ~6 Å [56]. The package of FebRNA can be available at https://github.com/Tan-group/FebRNA.

As described above, the knowledge-based fragment assembly model can efficiently predict RNA 3D structures with a relatively high accuracy. However, the performance of the models is strongly dependent on the quality and completeness of the template library and the performance for the structure evaluation method, in addition to secondary structures, which are required as the input. Although the number of RNA 3D structures deposited in the PDB database is continuously increasing, it may still be very difficult to find suitable templates for some special target RNAs, especially for multi-way junction templates, which are generally very crucial for building global RNA structures.

### 2.3. The Deep-Learning-Based Approaches

The advent of artificial intelligence has significantly advanced science and technology worldwide in recent years. A typical example is AlphaFold2, a deep-learning-based method for accurate protein 3D structure prediction [107,108,109]. However, it is essential to note that accurate prediction of macromolecule 3D structures generally requires extensive experimental structure data [107]. Based on the successful experience of deep learning in protein 3D structure prediction, some deep-learning-based methods have been developed to predict RNA 3D structures, although the available RNA 3D structures in the PDB database are rather limited compared with proteins [8]. In the following, we will introduce four deep-learning-based approaches for RNA 3D structure prediction developed very recently, which have been summarized in Table 3.

*RhoFold.* Shen et al. proposed an end-to-end deep-learning-based de novo RNA 3D structure prediction approach named RhoFold, which consists of three modules: structure feature extraction, structure prediction, and structure refinement [57]. The feature extraction module combines infernal and rMSA protocol to extract the MSA and pairwise residue features [57]. The structure prediction module, which is the core of RhoFold, predicts the rotation and translation matrices of the main frames based on the sequence and pair presentation from the feature extraction module. Finally, the structure refinement module modifies possible remaining structural conflicts, and the predicted structure is further relaxed through a constraint energy minimization algorithm. The prediction performance of RhoFold has been demonstrated by a test against the non-redundant RNA-Puzzle test dataset, and the average RMSD is less than 4 Å [57]. Additionally, RhoFold can also achieve promising predictions for the 3D structure of RNA complexes. RhoFold is available at https://github.com/RFOLD/RhoFold.

*DeepFoldRNA.* DeepFoldRNA is a fully automated end-to-end deep-learning-based method for RNA 3D structure prediction, composed of two consecutive modules: a constraint generation module and a structure construction module [58]. In the constraint generation module, multiple sequence alignments (MSAs) of RNAs are collected by iteratively searching multiple nucleic acid sequence databases using rMSA [110]. Afterwards, a self-attention neural network is utilized to predict pairwise distances and inter-residue/backbone torsion angles, and the predicted geometric constraints are transformed into composite potentials using the negative logarithmic likelihood of loading probability prediction in the structure construction module. Through the limited-memory Broyden–Letcher–Goldfarb–Shanno (L-BFGS) minimization algorithm, DeepFoldRNA achieves the end-to-end prediction for RNA 3D structures [58]. DeepFoldRNA was tested against two independent benchmark datasets from Rfam families (105 RNAs) [111] and RNA-Puzzle experiments (17 RNAs), where DeepFoldRNA predicts the structures with a mean RMSD of ~3 Å [58]. DeepFoldRNA is available at https://zhanggroup.org/DeepFoldRNA.

*trRosettaRNA.* Feng et al. proposed trRosettaRNA [59], a deep-learning-based de novo approach for RNA 3D structure prediction through the transformer network. trRosettaRNA follows a two-step procedure of trRosetta [112,113]: the first step is to predict 1D and 2D geometric shapes with a transformer network [59], and the geometrics include 1D orientations and 2D contacts, distances, and orientations. The second step is to generate 3D structures through energy minimization. The prediction performance of trRosettaRNA was demonstrated on two independent datasets from RNA-Puzzle datasets (30 RNAs) and Rfam families (101 RNAs), and the mean RMSDs of the predicted structures by trRosettaRNA are ~6 Å and <~4 Å, respectively [59]. Notably, trRosettaRNA performs similarly to DeepFoldRNA [58] in the all-atom RMSD, but predicts more realistic side-chain atoms. trRosettaRNA is available at https://yanglab.nankai.edu.cn/trRosettaRNA/.

*epRNA.* Sha et al. developed a neural network Euclidean parametrization-based method (epRNA) to predict RNA 3D structures solely from sequences, using the state-of-the-art neural network architecture and symmetries [60]. epRNA utilizes the parameterization of Euclidean distance matrices [114] to enable the neural network to directly output the distances between all of the residues. Subsequently, the structure predicted by the neural network is converted into an all-atom structure using DMD with constraints [28,115]. It is noted that epRNA achieves high accuracy predictions on the 3D structures of RNAs of up to 100 nucleotides in length. epRNA is available at https://bitbucket.org/dokhlab/eprna-euclidean-parametrization-of-rna/src/master/.

The above deep-learning-based methods can achieve high-quality and efficient prediction for RNA 3D structures, as tested against the available structure datasets. However, the reliability and performance of a deep-learning-based method strongly relies on the number and the structure spectrum of known RNAs, and due to the limited RNA 3D structures in the PDB database, it would be a great challenge for a deep-learning-based method to make blind predictions for the target RNAs, whose structures do not reside in the known structure spectrum. Nevertheless, these deep-learning models provide an alternative way for modeling RNA 3D structures, in addition to the physics-based methods and traditional knowledge-based models.

## 3. RNA 3D Structure Evaluation

Generally, a predictive model for RNA 3D structures would generate a candidate ensemble with more than one candidate for a target RNA, and consequently, a high-quality 3D structure evaluation is critical for an RNA 3D structure prediction model [61,62]. Some evaluation methods have been involved in the existing RNA 3D structure prediction models, such as selecting the cluster centers as the representative structures using the clustering algorithm, or directly evaluating the structures by the energies obtained from a statistical potential/scoring function/force field, or combining the clustering algorithm and energy functions/force fields to select the representative near-native structures [26,35,36,41]. However, an efficient scoring function with a high performance is still lacking and consequently is definitely required for RNA 3D structure evaluation. In the last two decades, several statistical potentials/scoring functions have been proposed for RNA 3D structure evaluation, owing to the prior progress made for proteins [61,62], which can be classified into knowledge-based scoring functions/statistical potentials [63,64,65,66,67,68,69] and deep-learning-based scoring functions [70,71], and are summarized in Table 4.

### 3.1. Knowledge-Based Scoring Functions/Statistical Potentials

In principle, any kind of geometrical parameters, such as distances or angles between atoms/atom groups, which can be utilized to distinguish a native conformation from a decoy one can be adopted to derive a statistical potential/scoring function [61,120]. According to Boltzmann’s law, a general expression of a statistical potential/scoring function can be obtained as a function of the geometrical parameter s [116]:

(2) $$\Delta E\left(s\right)=-k_{B}T\ln\left[\frac{P^{\mathrm{obs}}\left(s\right)}{P^{\mathrm{ref}}\left(s\right)}\right],\;$$

where $k_{B}$ and T are the Boltzmann constant and the temperature in Kelvin, respectively. $P^{\mathrm{obs}}\left(s\right)$ and $P^{\mathrm{ref}}\left(s\right)$ are the probability of the geometrical parameter s in the native and reference states, respectively. As shown in Equation (2), the reference state and the geometrical parameter *s,* which may involve two or more atoms, are crucial for building statistical potentials, and the core difference between various statistical potentials is attributed to the choice of them.

The native state is the non-redundant native structure ensemble, while an ideal reference state is a conformation ensemble composed of a non-redundant and complete spectrum of conformations in phase space and without interactions between atoms [120]. Nevertheless, an ideal reference state could not be obtained. Thus, several simulated reference states have been proposed by various approximations, including averaging [121], quasi-chemical approximation [117], atom-shuffled [122], finite-ideal-gas [118], spherical-non-interacting [123], and random-walk-chain [119] reference states. Moreover, geometrical parameters can be the inter-atom contact, inter-atom distance, inter-atom angle, inter-block orientation, and so on [61].

After deriving a statistical potential, the total energy ΔE(S,C) for a conformation *C* of a given sequence S can be given by the following [116]:

(3) ΔE(S,C)=∑ΔE(s), 

where the summation is overall applicable items for the geometrical parameter *s* with the additive assumption for the statistical potential [116].

#### 3.1.1. Two-Body Distance-Dependent Statistical Potentials

*RASP.* Based on the averaging reference state [121], in which the distribution of different atom pair types is approximately represented by the distribution averaged over all of the atom pair types in native structures, RASP was derived by Capriotti et al. at both CG and all-atom (23 clustered atom types) levels, and the distance between atom pairs was considered as the geometrical parameter [63]. Capriotti et al. showed that RASP had a better performance than NAST [27], which is a nucleotide-level CG statistical potential composed of bond, angle, dihedral, and non-bond terms. The package of RASP can be found at http://melolab.org/webrasp/home.php.

*KB potential*. Based on the quasi-chemical approximation reference state in which the number of certain atom−pair types should be proportional to the molar fraction of the corresponding ones from the native structures [117], Bernauer et al. proposed the distance-dependent statistical potentials of the KB potential at both CG and all-atom levels [64]. As a result of adopting Dirichlet process mixture models, the KB potentials are fully differentiable, which makes them applicable for molecular dynamics simulations [64].

*DFIRE-RNA*. Zhang et al. proposed an all-atom (85 atom types) distance-dependent statistical potential of DFIRE-RNA based on the finite-ideal-gas reference state [65], in which the pair distribution function in ideal gas is used to simulate the atomic pair distribution of RNAs in the reference state [65,118]. In DFIRE-RNA, a dimension parameter *α* is involved, which can help to better match the spatial scale of physical models to that of a realistic RNA or protein system [62,118]. For the RNA-Puzzles dataset, DFIRE-RNA was shown to have a consistently better performance than 3dRNAscore, RASP, and Rosetta energy function, which was combined through a series of knowledge-based and classical physics energy terms [45,63,65]. In addition, the package of DFIRE-RNA can be downloaded at https://github.com/tcgriffith/dfire_rna.

*rsRNASP*. Tan et al. recently developed an all-atom (85 atom types) distance-dependent statistical potential of rsRNASP by distinguishing local-ranged and non-local-ranged interactions at the residue separation level [66]. The averaging [121] and random-walk-chain [119] reference states were applied for extracting local-ranged and non-local-ranged potentials, respectively. For two test datasets from various structure prediction models, including the RNA-Puzzles dataset, rsRNASP showed an overall superior performance over the existing traditional statistical potentials (RASP, 3dRNAscore, and DFIRE-RNA) and deep-learning-based scoring functions (RNA3DCNN and ARES), which will be introduced below [66]. The package of rsRNASP is available at: https://github.com/Tan-group/rsRNASP.

*cgRNASP*. For high efficiency and direct applicability to CG-based RNA 3D structure prediction models, a series of residue-separation-based CG statistical potentials at different CG levels were recently proposed by Tan et al., and three-bead cgRNASP (12 CG atom types) is regarded as being representative [67]. Compared with the all-atom rsRNASP, the local-ranged interaction in cgRNASP was involved more subtly and completely through explicitly adding the interactions between the nearest neighbor residues and between the next-nearest ones [67]. Compared with rsRNASP, cgRNASP can have a similarly good or slightly better performance for extensive test datasets, while cgRNASP is strikingly more efficient than all-atom potentials such as rsRNASP, 3dRNAscore, and DFIRE-RNA. cgRNASP can be directly applicable to some existing CG-based RNA 3D structure prediction models [67]. The package of cgRNASP can be downloaded at https://github.com/Tan-group/cgRNASP.

#### 3.1.2. Two-Body Distance-Dependent and Angle-Dependent Statistical Potentials

*3dRNAscore*. Different from the above-described distance-dependent potentials such as RASP and KB potential, 3dRNAscore is composed of the distance- and torsion angle-dependent potentials based on the averaging reference state [68]. In 3dRNAscore, 85 atom types and 7 torsion angle types were involved [68], and a weight factor was optimized by the decoys of four typical RNAs generated by 3dRNA to balance the contributions of the two kinds of energy terms [68]. The involvement of the dihedral-dependent potential in 3dRNAscore has been shown to improve the evaluation performance [68]. 3dRNAscore has been embedded in 3dRNA, and the standalone package of 3dRNAscore can be found at http://biophy.hust.edu.cn/new/resources/3dRNAscore.

#### 3.1.3. Four-Body Contact Statistical Potential

*RAMP.* For capturing higher-order interactions beyond two-body potentials, Masso developed a four-body contact potential of RAMP, which is the first multi-body statistical potential for RNA 3D structure evaluation [69]. In RAMP, atomic four-body nearest-neighbors were divided by the Delaunary tessellation [124] for an RNA 3D structure, and each RNA was represented by four atom types of C, N, O, and P [69]. Thus, 35 distinct quadruplet types can be produced by the four-letter atomic alphabet. However, overall, RAMP has a worse performance than RASP-ALL when identifying native structures [69], which might be attributed to the fact that RAMP is a coarse-grained-level contact potential with only four clustered atom types, while RASP-ALL is a distance-dependent potential with 23 clustered atom types [61].

The further development of knowledge-based scoring functions/statistical potentials would benefit from the continuously increasing experimental structures deposited in the PDB database, which are currently inadequate. Moreover, the reference state and the geometrical parameter are crucial for building a knowledge-based scoring function. Thus, expanding the native structure database, modeling more realistic reference states for RNAs, and exploring unique RNA structure features as geometrical parameters could help improve the performance of a statistical potential/scoring function.

### 3.2. Deep-Learning-Based Scoring Functions

Beyond the above-described traditional statistical potentials/scoring functions, deep-learning techniques have recently been employed to develop scoring functions for RNA 3D structure evaluation. Generally, to develop a deep-learning-based scoring function does not require an artificial definition for RNA structure-related features or an explicit involvement of the reference state [70,71].

*RNA3DCNN.* Li et al. employed 3D convolutional neural network (3DCNN) to develop two 3DCNN-based scoring functions, named RNA3DCNN_MD and RNA3DCNN_MDMC, for assessing near-native RNA decoys and RNA decoys with large structure fluctuation, respectively [70]. 3DCNNs can directly use a 3D grid representation of RNA structures as the input without extracting RNA structure-related features manually, and the training sets of RNA3DCNN were generated by MD simulations and MC structure prediction for 414 RNAs [70]. RNA3DCNN_MD was trained by the first decoy set, while RNA3DCNN_MDMC was trained by the two decoy sets together [70]. For decoys with RMSDs less than 1.0 Å, RNA3DCNN was shown to have a similar or worse performance compared with the traditional statistical potentials (3dRNAscore, KB, RASP, and Rosetta), while for the RNA-Puzzles dataset, RNA3DCNN performed obviously better in identifying the native structures [70]. The package of RNA3DCNN can be downloaded at https://github.com/lijunRNA/RNA3DCNN.

*ARES.* Townshend et al. designed a neural network, the Atomic Rotationally Equivariant Scorer (ARES), to obtain the RMSDs of predicted structures from unknown native structures [71]. The initial layers of the ARES network with the 3D coordinates and chemical element type of each atom as the input were designed to recognize structural motifs, which were learned by training rather than being specified in advance. Furthermore, each layer of ARES was rotationally and translationally equivariant, which ensures that the corresponding transformation of its output could be achieved with the rotation or translation of its input. Thus, the orientation and position of an identified motif can be passed on to next layer of the network. Therefore, the initial layers of ARES can gather information locally, which can help to recognize finer-scale motifs (e.g., base pairs) and further recognize coarser-scale motifs (e.g., helices), and the remaining layers aggregate information across all atoms and capture the RNA global property, namely RMSD. Notably, the parameters of ARES were optimized with the training set, which contains decoy structures for 18 target RNAs generated by FARFAR2 [46]. ARES shows an excellent performance for evaluating structures from FARFAR2 [71], while becoming ordinary for the existing test datasets such as the RNA-Puzzles dataset [66,67]. ARES has been developed into a friendly webserver version: http://drorlab.stanford.edu/ares.html.

The deep-learning-based scoring functions could have outstanding performance in some aspects. For example, compared with previous statistical potentials/scoring functions, RNA3DCNN has an apparently better performance for identifying native/near-native structures [70], and ARES performs uniquely better for candidate structures from FARFAR2 [71]. Deep-learning-based scoring functions are generally free of artificially defined features and free of the reference states, which have natural advantages over the traditional statistical potentials/scoring functions [70,71]. However, the performance of a deep-learning-based scoring function is severely limited by its training dataset, such as the structure spectrum of the native structures and that of decoy structures. The incomplete spectrums of the native structures may lead to an unreliable performance of a scoring function for those structures not contributing to the native structure spectrum, and the spectrum of decoy structures may also cause a strong bias for the trained scoring function to the model for generating the decoy structures.

## 4. RNA 3D Structure Refinement

As described in Section 2 and Section 3, a predictive model for RNA 3D structures generally generates a structure ensemble through either force-field-guided conformation sampling of chains at different CG levels or structure assembly based on different-sized fragments [11,15]. Thus, a structure prediction model may produce many structure candidates with apparently abnormal structural domains containing imperfect stereochemistry information, such as unnatural bond lengths/angles and steric atom conflicts or incorrect tertiary interactions [72,75]. Afterwards, it is generally necessary to make structure refinement (optimization) for the identified (sampled/assembled) structures through a scoring function/evaluation algorithm. By combining the sampling algorithm (such as Gradient Descent, MC) with certain energy functions, the local and global structures of RNAs can be improved to their near-native states through adjusting the abnormal structure domains. Until now, most existing models for refining RNA 3D structures are physics-based ones [72,73,74,75], which are summarized in Table 5 and will be introduced below.

*QRNAS.* The model employs a modified version of Amber force field [76,77], incorporating four additional energy terms that are not explicitly present in the standard Amber force field, namely hydrogen bonds, base pair co-planarity, backbone regularization, and custom distance constraints [72]. By combining the fastest descent with golden section search and Polak−Ribiere conjugate gradient algorithm [125], QRNAS can significantly improve the local quality of RNA 3D structures while maintaining the global quality of RNA structures [72]. Additionally, QRNAS can refine not only RNA structures, but also DNA structures, chimeras, hybrids, and nucleic acids containing modified residues. QRNAS has been developed into a user-friendly software and is available at http://genesilico.pl/software/stand-alone/qrnas.

*BRiQ refinement.* BRiQ is a knowledge-based energy function at an atom level, which is corrected with quantum mechanics calculations on base−base interactions, and includes bonded and non-bonded terms [73]. The bonded term includes bond length, bond angle, torsion angle, and backbone rotameric interactions, and the non-bonded term comprises base−base, base−oxygen, oxygen−oxygen, and atomic clash interactions [73]. With utilizing the nucleobase-centric tree (NuTree) algorithm, BRiQ refinement achieves an atom-level refinement for RNA 3D structures, and the BRiQ refinement improves 81% Rosetta-SWM structures with RMSD < 2 Å, 100% RNA puzzle structures with RMSD < 4 Å, and 83% FARFAR2 structures with RMSD < 6 Å [73]. The package of BRiQ refinement is available at https://github.com/Jian-Zhan/RNA-BRiQ.

*RNAfitme.* Different from QRNAS [72] and BRiQ refinement [73], the RNAfitme model [74] refines RNA 3D structures by reconstructing them with fixed main chains and simulating them guided by the Charmm force field [79,80]. To refine an RNA structure with RNAfitme, five backbone atoms (O5′, C5′, C4′, C3′, and O3′) or nine backbone and sugar ring atoms (O5′, C5′, C4′, C3′, O3′, O4′, C1′, C2′, and O2′) need to be involved [74]. In the all-atom reconstruction process, the optimal fragments are selected using an adaptive matching algorithm, and a final all-atom RNA structure can be generated. Finally, NAMD [126] is used to relax the structure and reduce the spatial conflicts to improve the geometric shape of the preliminary RNA structure [74]. Although RNAfitme can hardly improve the global quality of an RNA structure, it can effectively solve the local spatial conflicts for RNA 3D structures. The web server of RNAfitme can be found at http://rnafitme.cs.put.poznan.pl/.

*3dRNA optimization.* Different from the above models [72,73,74], Wang et al. proposed a method to optimize the global quality of RNA 3D structures [75]. In this method, each nucleotide is represented by six CG beads at the atoms of P, C4′, C1′, C2, C4, and C6. The direct coupled analysis (DCA) [127,128] is used to extract evolutionary constraint information of RNA through multiple sequence alignments, and these constraints are complemented to a physics-based CG force field that includes bonded energies (bond length, bond angle, and dihedral angle) and non-bonded energies (base pairing, base stacking). Combining with the MC annealing algorithm, the method can be used to optimize an RNA 3D structure [75]. It is worth noting that the method can improve the global backbone structures of RNAs, especially for large RNA structures with tertiary contacts. The method has been embedded in 3dRNA and is available online at http://biophy.hust.edu.cn/new/3dRNA.

As described above, structure refinement would improve the local or global structures for RNAs. RNAfitme can reduce spatial conflicts in RNA structures [74], while compared with RNAfitme, QRNAS can also enforce backbone regularization and improve the base pair planarity [72]. However, RNAfitme and QRNAS can hardly improve the global quality of an RNA structure. BRiQ refinement can improve the base pairing structure and repair the RNA backbone structure, but it cannot make improvements for poor-quality RNA structures generated by structure prediction models. The 3dRNA optimization method [75] can significantly improve the backbone structure of large RNAs with tertiary contacts, while it may be difficult to achieve a high-precision RNA structure for those structures without tertiary contacts or no available DCA information. Thus, a high-precision refinement/optimization for RNA structures is still highly required to obtain high-precision RNA 3D structures.

## 5. Conclusions and Perspectives

Understanding the 3D structures of RNAs is crucial for unraveling the mysteries of the RNA world. As introduced above, a great progress has been made in modeling RNA 3D structures, including structure ensemble prediction, structure evaluation, and structure refinement. However, the existing models for RNA 3D structure prediction are still far away from ab initio predictions in terms of high accuracy solely from sequences, especially compared with protein 3D structure prediction. Here, we will discuss the major challenges and perspectives in modeling RNA 3D structures in different aspects.

### 5.1. On Physics-Based Structure Modeling

As introduced above, the existing physics-based models can make ab initio predictions for RNA 3D structures only relying on sequences. However, there are still challenges remaining for developing a reliable and applicable physics-based model.

First, the existing physics-based models are severely limited to the prediction accuracy and efficiency, and can generally be inapplicable for the ab initio structure predictions of large RNAs (e.g., >tens of nucleotides) and RNAs with complex topology such as multi-way junctions. The different-level CG approximations in the physics-based models would significantly reduce the structure representation complexity to improve simulation efficiency, while would also losing certain structure accuracy. Thus, achieving both high prediction accuracy and efficiency in CG representation modeling is an important challenge for physics-based RNA 3D structure modeling. Second, deriving an accurate force field/energy function is another critical challenge for a physics-based model to predict complex RNA 3D structures, especially for RNA structures with multi-way junctions and tertiary contacts. Third, developing an efficient and effective conformation sampling algorithm is important for a model to predict 3D structures of large RNAs as the employed simulated annealing, REMC, and REMD algorithms generally have huge computations despite their excellent ability to simulate structure folding on rugged energy landscapes. Fourth, a physics-based model essentially requires a high-quality rebuilding of all-atom structures due to the conventional CG simplification.

Therefore, multi-scale modeling may be very applicable for the further development of physics-based models, where a highly simplified CG-level representation would ensure the basic global structures and computation efficiency, and a final all-atom representation with an exact all-atom force field would ensure the predicted structure accuracy. Moreover, the direct involvement of a fragment assembly treatment after secondary structures fold may also be helpful for improving prediction efficiency for a physics-based model.

### 5.2. On Knowledge-Based Fragment-Assembly Structure Modeling

As described above, the fragment-assembly models are generally significantly more efficient than the physics-based models, while they are also limited to some severe challenges. First, the prediction accuracy of the existing fragment-assembly models is definitely limited to the quality of the input secondary structures. Thus, a blind prediction of such models would certainly require high-performance secondary structure prediction models. Second, the prediction performance severely depends on the completeness and quality of different types of RNA fragments, while, due to the limited RNA 3D structures in the PDB database, establishing a high-quality library of different types of fragments with a complete spectrum is impossible when only depending on the PDB database. Thus, it is very important to manually build the structures of core fragments, such as multi-way junctions and tertiary contacts for a fragment-assembly model. Third, this kind of model would generally generate structures with severe stereochemistry defects, such as abnormal backbone bond lengths/angles and severe atom conflicts, which require excellent structure refinement at the atom level.

Therefore, for this kind of model, (i) a benchmark survey may be first required on the existing secondary structure prediction methods in order for a blind prediction solely from sequences through a fragment-assembly model based on a more reliable secondary structures as an input, and (ii) combination with reliable physics-based models may be required to generate the 3D structures of core fragments that cannot be obtained through the RNA structures in the PDB database, and such combination may also help to diminish the severe stereochemistry defects in assembled 3D structures. 

### 5.3. On Deep-Learning-Based Structure Modeling

It has been shown that the existing deep-learning-based models are rather effective and efficient at predicting the known RNA 3D structures, while the models also have severe limitations.

First, the performance of deep-learning-based models is severely limited by the structure spectrum of the RNA structures in the PDB database. Due to possible “over-fitting” via the neural network, a deep-learning-based model may make accurate predictions for the RNA structures within the structure spectrum of training (known) structures, while it may completely fail to predict those outside of the training structure spectrum [129]. Second, the “black-box” training process makes a deep-learning-based model difficult to understand. Consequently, it becomes very hard to extend a deep-learning-based model in methodology rather than only extending training datasets. 

Therefore, a combination with physics-based models may be a possible way to reduce the effect of the ‘over-fitting’ training process and to improve the performance for deep-learning-based prediction, as a similar combination has shown its performance in RNA secondary structure modeling [130].

### 5.4. On Overall Modeling for RNA 3D Structures

In addition to the above-described specific limitations for different types of RNA 3D structure prediction models, there are still general challenges for modeling RNA 3D structures.

First, because of the highly polyanionic nature of RNAs, RNA 3D structures are strongly sensitive to the solution environment, such as ions, ligands, and other small molecules [131,132,133,134,135,136,137,138], while the existing structure prediction models rarely consider the effects of solution environments. Due to the critical role of ions in RNA structures and functions, it is still a challenge to properly involve the effects of ions in a structure prediction model, especially for the effect of Mg^2+^ in complex RNA structures [86,87]. Second, most existing structure prediction models are focused on static RNA 3D structures, while RNA functions can depend on proper structure changes upon the binding of other ions or ligands [139,140,141]. Thus, the knowledge of static 3D structures can sometimes become inadequate for understanding the functions of some RNAs, and it is still a challenge for a structure prediction model to predict RNA 3D structure change due to the interactions of ions/ligands/molecules or due to the change in temperature [15]. Third, non-canonical base pairs and nucleotide modifications such as m6A methylation and pseudouridylation are very important for RNA structures and functions [142,143], while it is still a challenge to predict RNA 3D structures with non-canonical base pairs and modified nucleotides. Fourth, the existing models essentially make in vitro structure predictions for RNAs, while cells can contain up to ~40% volume of various macromolecules such as proteins, DNA, and RNA [133,144,145]. The presence of crowding macromolecules can strongly affect the structures and stabilities of RNAs [135]. Thus, it is very necessary to make the predictions for RNA 3D structures in vivo, and it is still a challenge for structure prediction models to be able to involve the effect of in vivo. 

The effects of ions and temperature for complex RNA structures can be possibly captured in a multi-scale physics-based model with the involvement of applicable polyelectrolyte theories and experimental thermodynamic parameters, which has been shown by a CG physics-based model for predicting the thermal stability and ion effect for RNAs with simple topology such as hairpins and pseudoknots [33,35]. Moreover, the non-canonical base pairs and those with nucleotide modifications can be possibly involved in a physics-based structure prediction model through deriving the bond energy parameters for the base pairs based on the corresponding structures in the PDB database and using the corresponding Turner nearest-neighbor thermodynamic parameters [93]. Finally, the effect of an in vivo environment on RNA structures may be modeled based on an effective/efficient physics-based model by involving the effects of ions and temperature through explicitly including the crowders and the associated interactions [146].

### 5.5. On RNA 3D Structure Evaluation

Although important progress has been made in RNA 3D structure evaluation, there are several apparent limitations to the existing scoring functions. First, the performance of scoring functions is still apparently lower than satisfactory for realistic datasets such as the RNA-Puzzles dataset from various structure prediction models. For example, the Pearson correlation coefficients (PCC) between RMSDs and energies by the existing top scoring functions (e.g., rsRNASP [66], cgRNASP [67], DFIRE-RNA [65], and ARES [71]) are still less than 0.6 for the RNA-Puzzles dataset, a value far less than the ideal one of 1 [66,67]. Second, a structure prediction model can generate a huge number of structure candidates for structure evaluation. Thus, it is still a great challenge to develop a high-performance and efficient scoring function that is applicable to various structure prediction models. Third, the performance and applicability of a scoring function are also subject to the limited RNA 3D structures deposited in the PDB database. Thus, it is challenging to develop a high-performance scoring function based on an RNA structure database with an incomplete structure spectrum. Fourth, as RNA 3D structures can be strongly dependent on ion conditions and temperature, it is also necessary to develop a scoring function involving the effects of ion conditions and temperature to evaluate RNA structures at varying ion conditions and temperatures.

The further developments on a universal and efficient scoring function with a high performance may come from: (i) developing more realistic reference state or circumventing reference state, (ii) finding more proper geometrical parameters to better capture the relations between two atoms or among multiple atoms rather than the inter-atom distance, (iii) involving multi-body interactions beyond two-body potential, (iv) only keeping key (CG) atoms when developing scoring functions to improve the evaluation efficiency, and (v) specifically developing an effective and specific scoring function for a specific structure prediction model based on the training data from the model.

### 5.6. On RNA 3D Structure Refinement/Optimization

Although the existing structure refinement/optimization models can make the modeled RNA structures closer to their native states, they are also limited to some challenges. First, the existing models either mainly improve the local structures rather than the global structures for RNAs, or they improve the global structures rather than local structures for RNAs, and thus cannot effectively improve both the local and global structures. Second, a model for improving the both global and local structures at the atom level is computationally challenging [75]. 

Reliable structure refinement and optimization would significantly benefit structure prediction models as more near-native structures can be generated through the procedure. In this sense, a procedure of combining structure optimization with structure refinement may be applicable to obtain more accurate RNA 3D structures as the preceding one can generate more near-native global structures and the follow-up one can generate more local structures accurately. Moreover, a combination of a CG-level structure optimization and an atom-level refinement would also improve the computation efficiency of the procedure.

In summary, great progress has been made in the recent two decades in modeling RNA 3D structures, while many efforts are still required in order to archive accurate predictions for RNA 3D structures and to understand the associated RNA functions in vivo. Aided by the increase in RNA structure data and the advance in physics-based modeling techniques and computational technology, we anticipate exciting developments in modeling RNA 3D structures in the near future.

## Figures and Tables

**Table 1:** The existing physics-based CG models for RNA 3D structure prediction.

Models	Refs.	CG Beads	Sampling ^a^	Final Structures	From Sequence *^e^*	Availability
YUP	[25,26]	1-bead	MC	Lowest-energy structure ^b^	No	http://rumour.biology.gatech.edu/YammpWeb/
NAST	[27]	1-bead	MD	Centroid structures of clusters ^c^	No	https://simtk.org/home/nast
iFoldRNA	[28,29]	3-bead	DMD	Centroid structures of clusters ^c^	Yes	https://dokhlab.med.psu.edu/ifoldrna
CG model with salt effect	[30,31,32,33,34,35]	3-bead	REMC	Lowest-energy structure ^d^	Yes	No
SimRNA	[36,37]	5-bead	REMC	Centroid structures of clusters ^c^	Yes	https://genesilico.pl/SimRNAweb
IsRNA/IsRNA1	[38,39]	4/5-bead	REMD	Centroid structures of clusters ^c^	Yes	http://rna.physics.missouri.edu/IsRNA/index.html
IsRNA2	[40]	5-bead	REMD	Centroid structures of clusters ^c^	Yes	http://rna.physics.missouri.edu/IsRNA/index.html
RNAJP	[41]	5-bead	MD	Lowest-energy structure ^b^	No	http://rna.physics.missouri.edu/RNAJP/index.html
HiRE-RNA	[42]	6/7-bead	REMD	Centroid structures of clusters ^c^	Yes	No
Ernwin	[43]	helix-centered	MCMC	Lowest-energy structure ^b^	No	http://github.com/pkerpedjiev/ernwin

^a^ MC, MD, DMD, REMC, REMD, and MCMC represent Monte Carlo, molecular dynamics, discrete molecular dynamics, replica exchange Monte Carlo, replica exchange molecular dynamics, and Markov chain Monte Carlo simulation, respectively. ^b^ The lowest-energy structures are determined by the energy functions from the corresponding prediction models. ^c^ The centroid structures of clusters are obtained through clustering the low-energy structures by the energy functions from the corresponding prediction models. ^d^ The lowest-energy structures are determined by a knowledge-based statistical potential of cgRNASP [67]. *^e^* Can the model make predictions solely from sequence?

**Table 2:** The existing knowledge-based fragment assembly models for RNA 3D structure prediction.

Models	Refs.	Fragment Feature	Final Structures	Availability
FARNA/FARFAR2	[44,45,46]	3-nucleotide fragments	Centroid structures of clusters ^c^	https://rosie.rosettacommons.org/farfar2
MC-Fold/MC-Sym	[47]	SSE	Lowest-energy structures ^d^	http://www.major.iric.ca
RNAComposer	[48,49]	SSE	The representative structure is assembled from the best templates	http://rnacomposer.ibch.poznan.pl
3dRNA	[50,51,52,53]	SSE ^a^	Lowest-energy structures ^e^	http://biophy.hust.edu.cn/new/3dRNA
Vfold3D	[54]	CG SSE ^b^	The representative structure is assembled from the best templates	http://rna.physics.missouri.edu/vfold3D/
VfoldLA	[55]	SSE ^a^	Centroid structures of clusters	http://rna.physics.missouri.edu/vfoldLA/
FebRNA	[56]	CG SSE ^b^	Lowest-energy structure ^f^	https://github.com/Tan-group/FebRNA

The smallest secondary elements (SSE) are defined as base pair, hairpin loop, internal loop, bulge loop, pseudoknot loop, and junction. ^a^ SSEs contain additional base pairs at their ends. ^b^ Coarse-grained (CG) SSEs contain base pairs at their ends. ^c^ Centroid structures of clusters are determined through clustering the low-energy structures by the energy functions from the corresponding prediction models. ^d^ The lowest-energy structures are determined by Amber’99 force field [102]. ^e^ The lowest-energy structures are determined through clustering the assembled structures and ranking the cluster centers using 3dRNAscore [68]. ^f^ The lowest-energy structures are determined by a knowledge-based statistical potential [67].

**Table 3:** The existing deep-learning-based approaches for RNA 3D structure prediction.

Approaches	Refs.	Neural Network Learning Information	Final Structures	Availability
RhoFold	[57]	Sequence representations and interactions between different nucleotides	Lowest-energy structure	https://github.com/RFOLD/RhoFold
DeepFoldRNA	[58]	Structural information from evolutionary profiles	Lowest-energy structure	https://zhanggroup.org/DeepFoldRNA
trRosettaRNA	[59]	MSA and secondary structure representations	Lowest-energy structure	https://yanglab.nankai.edu.cn/trRosettaRNA/
epRNA	[60]	RNA sequences	Centroid structures of clusters	https://bitbucket.org/dokhlab/eprna-euclidean-parametrization-of-rna/src/master/

**Table 4:** The existing scoring functions/statistical potentials for RNA 3D structure evaluation.

Knowledge-Based Scoring Functions					
Scoring Functions	Refs.	Reference States	Geometrical Parameters	Atom Types	Availability
RASP-ALL	[63]	Averaging [116]	Distance between atom pairs	23	http://melolab.org/webrasp/home.php
All-atom KB potential	[64]	Quasi-chemical approximation [117]	Distance between atom pairs	85	No
DFIRE-RNA	[65]	Finite-ideal-gas [118]	Distance between atom pairs	85	https://github.com/tcgriffith/dfire_rna
rsRNASP	[66]	Averaging [116] + Random-walk-chain [119]	Distance between atom pairs	85	https://github.com/Tan-group/rsRNASP
cgRNASP	[67]	Averaging [116] + Finite-ideal-gas [118]	Distance between atom pairs	12	https://github.com/Tan-group/cgRNASP
3dRNAscore	[68]	Averaging [116]	Distance between atom pairs and torsional angles of backbone	85	http://biophy.hust.edu.cn/new/resources/3dRNAscore
RAMP	[69]	Multinomial reference distribution	Atomic quadruplet interaction	4	No
**Deep-Learning-Based Scoring Functions**					
**Scoring Functions**	**Refs.**	**Reference States**	**Geometrical Parameters**	**Atom Types**	**Availability**
RNA3DCNN	[70]	Free	Free, and the 3D grid representation of RNA structure as the input	85	https://github.com/lijunRNA/RNA3DCNN
ARES	[71]	Free	Free and the 3D coordinates and chemical element type of each atom as the input.	85	http://drorlab.stanford.edu/ares.html

**Table 5:** The existing physics-based approaches for RNA 3D structure refinement.

Approaches	Refs.	Force Field	Refinement Characteristics	Availability
QRNAS	[72]	Amber with four optional energy terms	Reducing clash, enforcing backbone regularization, explicit hydrogen bonds, base pair co-planarity. co-planarity	http://genesilico.pl/software/stand-alone/qrnas
BRiQ refinement	[73]	A fully knowledge-based atom-level force filed	Reducing clash, improving base pairing and backbone structures	https://github.com/Jian-Zhan/RNA-BRiQ
RNAfitme	[74]	Charmm force field	Reducing clash and smoothing the structure	http://rnafitme.cs.put.poznan.pl/
3dRNA optimization	[75]	CG force field with evolutionary restraints from DCA	Improving global backbone structure	http://biophy.hust.edu.cn/new/3dRNA

## Data Availability

The data presented in this review come from the corresponding references.
